# Interrelationship between *TP53 *gene deletion, protein expression and chromosome 17 aneusomy in gastric adenocarcinoma

**DOI:** 10.1186/1471-230X-9-55

**Published:** 2009-07-20

**Authors:** André S Khayat, Adriana C Guimarães, Danielle Q Calcagno, Aline D Seabra, Eleonidas M Lima, Mariana F Leal, Mário HG Faria, Silvia HB Rabenhorst, Paulo P Assumpção, Samia Demachki, Marília AC Smith, Rommel R Burbano

**Affiliations:** 1 Human's Cytogenetics Laboratory, Institute of Biological Sciences, Federal University of Pará, Av Augusto Correa 01, 66075-900, Belém, PA, Brazil; 2 Biology Department, Campus Ministro Reis Velloso/Parnaíba, Federal University of Piauí, PI, Brazil; 3 Genetics Division, Department of Morphology, Federal University of São Paulo, São Paulo, SP, Brazil; 4 Molecular's Genetics Laboratory, Department of Pathology, Medical School, Federal University of Ceará, Fortaleza, CE, Brazil; 5 João de Barros Barreto University Hospital, Federal University of Pará, Belém, PA, Brazil

## Abstract

**Background:**

This study evaluates the existence of numerical alterations of chromosome 17 and *TP53 *gene deletion in gastric adenocarcinoma. The p53 protein expression was also evaluated, as well as, possible associations with clinicopathological characteristics.

**Methods:**

Dual-color fluorescence *in situ *hybridization and immunostaining were performed in twenty gastric cancer samples of individuals from Northern Brazil.

**Results:**

Deletion of *TP53 *was found in all samples. *TP53 *was inactivated mainly by single allelic deletion, varying to 7–39% of cells/case. Aneusomy of chromosome 17 was observed in 85% of cases. Chromosome 17 monosomy and gain were both observed in about half of cases. Cells with gain of chromosome 17 frequently presented *TP53 *deletion. The frequency of cells with two chr17 and one *TP53 *signals observed was higher in diffuse than in intestinal-type GC. Immunoreactivity of p53 was found only in intestinal-type samples. The frequency of cells with two chr17 and two *TP53 *signals found was higher in samples with positive p53 expression than in negative cases in intestinal-type GC.

**Conclusion:**

We suggest that *TP53 *deletion and chromosome 17 aneusomy is a common event in GC and other *TP53 *alterations, as mutation, may be implicated in the distinct carcinogenesis process of diffuse and intestinal types.

## Background

Gastric cancer (GC) is the fourth most frequent malignancy and the second most common cause of cancer death in the World [1]. In the State of Pará (Northern Brazil), GC was the most common cause of cancer death in 2000. In Belém, State of Pará, the 5-year-survival rate is about 9–10% [2]. A better understanding of the biology of this neoplasia progression is crucial for the development of better tests to early neoplasia detection and also of new treatment strategies for GC.

Molecular events in the carcinogenesis of GC remain largely unknown [3]. A key feature in the pathogenesis of most GC, as in many other solid cancers, is chromosomal instability, resulting in gains and losses of parts or even whole chromosomes [4].

Gastrointestinal tract tumors are notorious for being difficult to be analyzed by standard cytogenetic techniques [5-9]. Fluorescence *in situ *hybridization (FISH) assay allows rapid detection of numerical genetics aberrations in interphase nuclei in tumor cells. FISH assay should be used to evaluate cell-to-cell heterogeneity in gene or loci copy number and detect small subpopulations of genetically aberrant cells [10]. FISH studies have shown numerical aberrations 1, 7, 8, 9, 17, 20, X and Y to be common in GC [[7,11-19], see also review [20]]. There are some studies in literature concerning *TP53*, located at chromosome 17p13.1, and chromosome 17 (chr17) copy number alterations by FISH assay in GC [[21-24], see also review [20]].

The *TP53 *tumor suppressor plays a pivotal role in the coordination of the repair process or in the induction of apoptosis. *TP53 *somatic alteration is described in approximately 50% of human cancers, including GC [25]. Deregulation of the *TP53 *pathway has been shown to involve mutations, loss of heterozygosity (LOH), increased expression of the *TP53 *inhibitor HDM2, or epigenetic silencing of the *TP53 *promoter [26,27].

The aim of this study was to investigate chr17 and *TP53 *numerical alterations in GC samples from Pará State by dual-color FISH technique. Immunostaining for p53 protein was also evaluated. These results were correlated with clinicopathological characteristics.

## Methods

### Samples

The study included 20 gastric adenocarcinoma samples. Samples of primary tumors submitted to surgical resection were obtained from João de Barros Barreto University Hospital (HUJBB). This study investigated cancer samples of patients from Pará State, where there is a mixed population composed of three main ethnic groups: Amerindian, African and European [28].

Patients' age, sex and tumor anatomical sites were obtained from tumor registries. The mean age of the twenty patients was 55 ± 14.67 years (range 24–77). The female/male ratio was 3:2. All samples were classified according to Laurén [29] and tumors were staged using standard criteria by TNM staging [30]. According to Laurén's classification, 6 were diffuse type (30%) and 14 were intestinal type (70%). Table 1 shows cases with their histopathological characteristics.

**Table 1:** Clinicopathological, immunohistochemistry and FISH results of GC samples.

							FISH chr17/*TP53 *(%)													
							
Case	Age	Sex	Loc^†^	pTNM^‡^	LC^§^	IHC	2/2	2/1	1/1	3/2	3/1	3/3	4/2	4/1	1/2	2/3	4/4	4/3	5/4	2/4
1	77	M	An	T2N1Mx	Int	+	77.5	11.0	10.0	1.0	-	0.5	-	-	-	-	-	-	-	-
2	48	F	An	T4N0Mx	Int	-	52.2	16.0	4.1	1.2	2.4	0.6	8.2	-	-	-	13.5	1.8	-	-
3	58	M	Co/an	T1N1Mx	Int	+	68.5	14.5	4.5	-	3.0	-	3.5	2.5	1.5	0.5	-	1.5	-	-
4	48	F	Co/an	T3N0Mx	Int	+	62.2	15.6	10.0	3.2	0.5	-	6.5	1.0	-	0.5	0.5	-	-	-
5	24	F	Co/an	T3N3Mx	Int	-	50.7	39.0	9.6	-	-	-	-	-	0.7	-	-	-	-	-
6	71	F	An/py	T2N0Mx	Dif	-	65.0	20.0	12.0	1.0	-	2.0	-	-	-	-	-	-	-	-
7	41	F	Co/an/py	T4NxMx	Int	+	84.5	7.8	6.5	-	-	0.6	0.6	-	-	-	-	-	-	-
8	63	F	Co/an	T4N3M1	Dif	0	67.0	25.0	3.0	2.0	-	1.0	-	-	-	-	1.5	-	-	0.5
9	68	F	An	T1N1Mx	Int	+	70.0	11.5	4.5	3.0	2.0	-	4.0	1.5	1.0	0.5	2.0	-	-	-
10	76	M	An/py	T3N1M1	Int	-	68.0	14.0	2.0	2.0	6.0	-	5.0	0.5	2.5	-	-	-	-	-
11	41	F	All	T3N1M0	Dif	-	54.5	29.5	-	2.0	3.5	-	9.0	0.5	0.5	0.5	-	-	-	-
12	60	M	An	T3N2Mx	Int	0	88.0	7.0	5.0	-	-	-	-	-	-	-	-	-	-	-
13	65	M	Ca/fu	T3N1Mx	Int	0	69.0	16.0	10.5	-	0.5	2.0	1.0	0.5	-	-	-	-	0.5	-
14	52	M	Co/ca	T2N1Mx	Int	+	76.5	15.0	7.5	-	-	0.5	0.5	-	-	-	-	-	-	-
15	48	M	Co/an	T2N1Mx	Intl	+	54.0	24.0	1.5	5.0	5.5	2.5	6.0	1.5	-	-	-	-	-	-
16	52	F	An/py	T3N1Mx	Int	-	53.4	27.0	1.6	3.2	4.2	1.6	8.0	-	-	1.0	-	-	-	-
17	50	F	An/py	T4N1Mx	Int	+	63.0	19.0	4.0	2.0	0.5	1.0	7.5	-	2.0	1.0	-	-	-	-
18	47	F	An	T2N0Mx	Dif	-	65.0	17.5	9.5	1.0	2.5	1.5	1.5	1.0	-	-	0.5	-	-	-
19	74	F	Co/an/py	T4N1Mx	Dif	-	67.0	21.5	-	1.0	7.5	-	1.5	1.5	-	-	-	-	-	-
20	31	M	Co/an/py	T3N1Mx	Dif	-	62.7	24.3	2.7	5.5	1.6	-	2.7	0.5	-	-	-	-	-	-

† Tumor location: An = antrum; Ca = cardia; Co = corpus; Fu = fundus; Py = pylorus | ‡ TNM pathological staging | §Laurén's classification: Int = Intestinal; Dif = Diffuse | Immunostaining: 0 = not done; (-) without immunoreactivity; (+) with immunoreactivity.

All patients had negative histories of exposure to either chemotherapy or radiotherapy prior to surgery; there was no other diagnosed cancer. An informed consent with approval of the ethics committee of HUJBB was obtained from the studied patients.

### Fish

FISH was applied on cells fixed in methanol/acetic acid using recently made slides according to modified protocols [31]. The slides were washed in 2× saline sodium citrate (SSC)/0.5% NP-40 (pH 7.0) solution and dehydrated in 70%, 80% and 95% ethanol. To determine the chr17 and *TP53 *copy numbers, cells were hybridized with 10 μL dual-color direct labeled probe (Qbiogene^®^, CA, USA) specific for chr17 α-satellite and *TP53 *gene region, labeled with fluorescein and rhodamine respectively. The probe applied to the slide under a glass coverslip. The probe and sample were denatured at 75°C for 5 minutes and. *In situ *hybridization occurred at 37°C in a moist chamber overnight. Post-hybridization washings were done and the nuclei were counterstained with DAPI/antifade. Molecular cytogenetic analysis was carried out under an Olympus BX41 fluorescence microscope with triple DAPI/FITC/TRICT filter (Olympus, Japan) and the FISHView^® ^of Applied Spectral Imaging^® ^image analysis system (ASI Ldt., Israel). For each case, 200 interphase/metaphase nuclei were analyzed and were scored using the Hopman's criteria [32]. In our study, the cut-off level for interphase-FISH was 5%. To avoid misinterpretation due to technical error, gastric mucosal tissue (nonneoplastic) and normal lymphocyte nuclei were used as negative control.

### Immunohistochemical staining

Deparaffinized tissue sections (4 μm) were incubated with primary monoclonal antibody p53 (DO-7, dilution 1:50, DakoCytomation, CA, USA) and secondary antibody followed by streptavidin-biotin-peroxidase complex (DakoCytomation, CA, USA) as previously described [18]. Slides were visualized with diaminobenzidine-H_2_O_2 _and counterstained with Harry's hematoxylin. The results were interpreted using the Ozturk's et al. criteria [33]. Positive p53 expression was defined as clear nuclear staining, whereas negative p53 immunostaining was considered when no positive cell was seen or rare cells were stained (less than 10% weakly stained tumor cells). A breast adenocarcinoma sample with known p53 immunoreactivity was used as positive control and a normal gastric mucosa as negative control. Two pathologists evaluated the immunostaining results independently.

### Statistical analyses

Statistical analyses were performed using Fisher's exact test and Mann-Whitney test. P value < 0.05 was considered to be statistically significant.

## Results

### FISH

Lymphocyte nucleus and normal gastric mucosa showed two signals to chr17 and *TP53 *in 97.5% and 96.5% of analyzed cells respectively. All cancer samples presented numerical alterations of chr17 and *TP53 *gene. Normal nuclei were observed in 50.7–88% of cells/case (Table 1).

**Figure 1:**
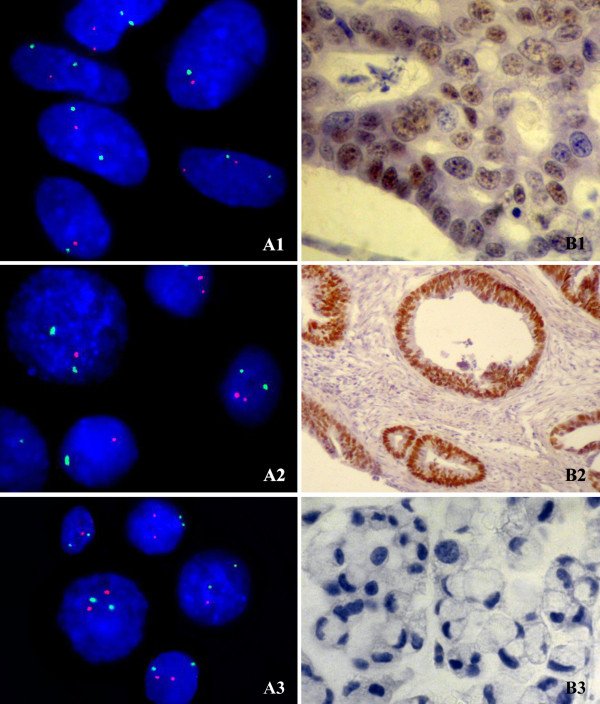
**Cells submitted to FISH (A) and immunohistochemistry (B) assays**. A1 (case 14), A2 (case 5) and A3 (case 6): interphase nuclei presenting chr17 monosomy (green signal) with one copy of *TP53 *(red signal), and nuclei presenting chr17 disomy with one or two copies of *TP53 *– 1000× magnification; B1 (case 14) and B2 (case 17): tissue with nuclear p53 immunoreactivity (brown stain) – 400× and 100× magnification, respectively; B3 (case 6): tissue without p53 immunoreactivity – 400× magnification.

The main *TP53 *alteration observed was the single allelic deletion. This alteration was present in all cases, varying to 7–39% of cells/case (Figure 1A). Chr17 monosomy observed in 45% of samples, ranging 5–12% of cells/case. Chr17 gain was also detected in 45% of cases. Chr17 trisomy was observed in 20% of the cases in a frequency up to 7.5% cells/case (case 19), in which 40% and 60% of these cases showed two and one single *TP53 *copy number respectively. Chr17 tetrasomy with two *TP53 *signals was detected in 35% of cases in a frequency up to 9% cells/case. Four signals to chr17 and *TP53 *was observed in one case (case 2) in 13.5% of cells (Table 1).

The frequency of cells with two chr17 and one *TP53 *signals observed was higher in diffuse than in intestinal-type GC (p = 0.026). Chromosome alterations were not associated with other clinicopathological characteristics.

### p53 protein expression

In the present study, breast adenocarcinoma (positive control) showed p53 overexpression and the normal gastric mucosa (negative control) showed lack of p53 immunoreactivity.

Seventeen GC samples were analyzed by immunostaining. Immunoreactivity of p53 (positive) was found in 8 cases (47%) (Figure 1B). All of these cases were intestinal-type (8 of 12 samples). Statistical analysis showed an association between intestinal-type GC and p53 expression (p = 0.0294). In our sample, p53 immunoreactivity was not associated with age, gender, location and TNM status (p > 0.05) (Table 1).

The frequency of cells with two chr17 and one *TP53 *signals observed was higher in samples with immunoreactivity of p53 negative than in cases with positive p53 expression (p = 0.016). In intestinal-type GC, the frequency of cells with two chr17 and two *TP53 *signals found was higher in samples with positive p53 expression than in negative cases (p = 0.027).

## Discussion

Aneusomy is one of the most common findings in human cancer. Chromosome copy number changes encompass a continuum ranging from sporadic events to a change of chromosome numbers with each cell division. Although aneusomy can be detected at early stages of transformation and even in certain premalignant lesions, the degree of numerical chromosomal aberrations generally increases with tumor progression, and tumors with aggressive clinical behavior are more likely to be aneusomy than less malignant tumors. Aneusomy has also been found to be associated with poor treatment outcome in cancer patients [34].

Regarding chr17 and *TP53 *copy number, we observed normal nuclei in 50.7–88% of cells/case. This result corroborates our previous conventional and molecular cytogenetic studies, which demonstrated that chr17 aneusomy is not a frequent in GC samples of individual from Northern Brazil [7,35,36].

There are few studies in literature concerning chr17 and *TP53 *copy number alterations. Our findings corroborated Kobayashi et al. [21] that found deletion of *TP53 *in 39% of 67 tumors and all of these samples showed chr17 polysomy. Takahashi et al. [22] also observed that p53 signal count was lower than the chromosome 17 signal count in 1 of 3 intestinal-type GC.

Gomyo et al. [23] demonstrated 3 or 4 signals for chr17 in 46% of 13 intestinal-type GC samples and 77% of these cases showed *TP53 *deletion by FISH assay. In our sample, 45% of all cases presented 3 or 4 signals for chr17 and *TP53 *deletion was detected in all cases.

Suzuki et al. [24] observed an increased of chr17 polysomy frequency and the incidence of *TP53 *deletion ranged from 55% to 90% in ten GC samples. They also described that *TP53 *deletion was significantly higher in intestinal than in diffuse-type cancers. However, in our sample we found *TP53 *deletion in up to 49% cells/case and the frequency of cells with two chr17 and one *TP53 *signals observed was higher in diffuse than in intestinal-type GC. Inconsistencies regarding the frequency of *TP53 *deletion in GC between our study and Suzuki et al. [24] may be suggestive of distinct gastric carcinogenesis pathways in different ethnic composition or differences in stage when the analysis was done. It is widely reported that differences between carcinogenesis processes can be the result of distinct environmental and genetic factors.

Suzuki et al. [24] also observed that chr17 monosomy was present in 70% of 10 cases and the most frequent pattern in these cases was the combination of one copy of chr17 and one of *TP53*. On the other hand, in our sample we observed chr17 monosomy in 45% of 20 cases (cut-off level of 5%) and the more frequent pattern was the combination of two copies of chr17 with one *TP53 *copy by cell.

In the present study, chr17 tetrasomy with two *TP53 *signals was frequently observed. We also could observe that chr17 gain tended to be more frequently found in tumors with higher extension (T3 or T4 stages). This finding suggests that tetrasomy event is a subsequent step after gene deletion, which could justify the higher frequency of cells with two copies of chr17 and one *TP53 *copy and also the tendency of increased level of chr17 gain in tumors with higher extension. Galipeau et al. [37] suggested that increased polysomy level is associated with inactivation of the *TP53 *in Barrett's esophagus in vivo, supporting our hypothesis.

Williams et al. [38] described that *TP53 *deletion was the most common aberration in gastritis, intestinal metaplasia, dysplasia e GC by FISH assay. The author suggested that this abnormality may exist in the initiation and progression to gastric cancer.

*TP53 *deletion, as well as chromosome 17 aneusomy, was observed in all analyzed samples, despite Laurén's histopathologic types. However, differential p53 expression was detected between these groups.

Increased immunostaining of p53 can depend on either increased synthesis of wild-type protein or accumulation of mutated protein in the cell, since the antibody recognizes both types of the protein [39]. In the present study, we observed an increased frequency of immunostained nuclei and the greater staining intensity in 47% of GC samples, as compared to normal gastric mucosa. We suggest that the p53 overexpression may be related to the mutated type of this protein. The frequency of p53 overexpression in GC has been described varying from 19% to 57.5% of cases [23,40-44] and some studies also described *TP53 *mutations related with its protein overexpression [23,41].

In the present study, only intestinal-type GC presented p53 immunoreactivity. Our research suggests that, beside *TP53 *loss by allelic deletion or chr17 aneusomy, a mutation in the remaining *TP53 *allele may exist in intestinal-type GC samples, which would explain the protein immunoreactivity. On the other hand, two possibilities might be considered to the absence of immunoreactivity in diffuse-type GC: this absence was not due to mutations in *TP53 *gene or an eventual mutation in this gene would not interfere in the protein accumulation. In both situations the immunoreactivity cannot be detected.

The p53 expression was also associated with a higher frequency of cells with two chr17 and two TP53 signals in intestinal-type GC. We hypothesize that these cells may present TP53 with mutations and this event could be occurring earlier than allelic deletion in intestinal-type gastric carcinogenesis. Further investigations concerning *TP53 *mutations and expression should be done in larger samples, also including early GC specimens.

## Conclusion

Our findings showed that *TP53 *deletion and chromosome 17 aneusomy are common events in GC. Our results also suggest that LOH is an important *TP53 *alteration in GC. However, other *TP53 *alterations than allelic deletion may be implicated in the carcinogenesis process.

## Competing interests

The authors declare that they have no competing interests.

## Authors' contributions

RRB and MACS designed the study. ASK, ACG, DQC, ADS, EML, MFL were involved in data collection, literature searches, genetic and statistical analysis. MHGF, SHBR, SD were involved in pathological analysis. PPA recruited patients and was responsible by samples collection. ASK wrote the first draft of the manuscript. All authors listed have contributed to all subsequent drafts, and have approved the final manuscript.

## Pre-publication history

The pre-publication history for this paper can be accessed here:

http://www.biomedcentral.com/1471-230X/9/55/prepub

## References

[B1] Parkin DM Bray F Ferlay J Pisani P Global cancer statistics, 2002 CA Cancer J Clin 2005 55 74 108 10.3322/canjclin.55.2.74 15761078

[B2] Resende ALS, Mattos IE, Koifman S Mortalidade por Câncer Gástrico no Estado do Pará, 1980–1997 Arq Gastroenterol 2006 43 DATASUS/Ministério da Saúde, Brasil, Informações de saúde 247 52 17160244 10.1590/s0004-28032006000300018

[B3] Kimura Y Noguchi T Kawahara K Kashima K Daa T Yokoyama S Genetic alterations in 102 primary gastric cancers by comparative genomic hybridization: gain of 20q and loss of 18q are associated with tumor progression Mod Pathol 2004 17 1328 37 10.1038/modpathol.3800180 15154013

[B4] Lengauer C Kinzler KW Vogelstein B Genetic instabilities in human cancers Nature 1998 396 643 9 10.1038/25292 9872311

[B5] Ferti-Passantonopoulou AD Panani AD Vlachos JD Raptis SA Common cytogenetic findings in gastric cancer Cancer Genet Cytogenet 1987 24 63 73 10.1016/0165-4608(87)90083-5 3791173

[B6] Xia JC Lu S Geng JS Fu SB Li P Liu QZ Direct chromosome analysis of ten primary gastric cancers Cancer Genet Cytogenet 1998 102 88 90 10.1016/S0165-4608(97)00293-8 9530349

[B7] Assumpção PP Ishak G Chen ES Takeno SS Leal MF Guimarães AC Calcagno DQ Khayat AS Demachki S Smith Mde A Numerical aberrations of chromosome 8 detected by classic cytogenetic and Fluorescence in situ Hybridization in individuals from Northern Brazil with gastric adenocarcinomas Cancer Genet Cytogenet 2006 169 45 9 10.1016/j.cancergencyto.2006.03.019 16875936

[B8] Ochi H Douglass HO Jr Sandberg AA Cytogenetic studies in primary gastric cancer Cancer Genet Cytogenet 1986 22 295 307 10.1016/0165-4608(86)90022-1 3731046

[B9] Kitayama Y Igarashi H Sugimura H Different vulnerability among chromosomes to numerical instability in gastric carcinogenesis: stage-dependent analysis by FISH with the use of microwave irradiation Clin Cancer Res 2000 6 3139 46 10955795

[B10] Kallioniemi A Visakorpi T Karhu R Pinkel D Kallioniemi OP Gene Copy Number Analysis by Fluorescence in Situ Hybridization and Comparative Genomic Hybridization Methods 1996 9 113 21 10.1006/meth.1996.0015 9245350

[B11] Panani AD Ferti AD Avgerinos A Raptis SA Numerical aberrations of chromosome 8 in gastric cancer detected by fluorescence in situ hybridization Anticancer Res 2004 24 155 9 15015591

[B12] van Dekken H Pizzolo JG Kelsen DP Melamed MR Targeted cytogenetic analysis of gastric tumors by in situ hybridization with a set of chromosome-specific DNA probes Cancer 1990 66 491 7 10.1002/1097-0142(19900801)66:3<491::AID-CNCR2820660315>3.0.CO;2-Q 2364362

[B13] Han K Oh EJ Kim YS Kim YG Lee KY Kang CS Kim BK Kim WI Shim SI Kim SM Chromosomal numerical aberrations in gastric carcinoma: analysis of eighteen cases using in situ hybridization Cancer Genet Cytogenet 1996 92 122 9 10.1016/S0165-4608(96)00165-3 8976368

[B14] Beuzen F Dubois S Flejou JF Chromosomal numerical aberrations are frequent in oesophageal and gastric adenocarcinomas: a study using in-situ hybridization Histopathology 2000 37 241 9 10.1046/j.1365-2559.2000.00887.x 10971700

[B15] Fringes B Mayhew TM Reith A Gates J Ward DC Numerical aberrations of chromosomes 1 and 17 correlate with tumor site in human gastric carcinoma of the diffuse and intestinal types. Fluorescence in situ hybridization analysis on gastric biopsies Lab Invest 2000 80 1501 8 11045566 10.1038/labinvest.3780159

[B16] Kitayama Y Igarashi H Watanabe F Maruyama Y Kanamori M Sugimura H Nonrandom chromosomal numerical abnormality predicting prognosis of gastric cancer: a retrospective study of 51 cases using pathology archives Lab Invest 2003 83 1311 20 10.1097/01.LAB.0000087622.80751.C5 13679439

[B17] Calcagno DQ Leal MF Taken SS Assumpção PP Demachki S Smith MA Burbano RR Aneuploidy of chromosome 8 and *C-MYC *amplification in individuals from northern Brazil with gastric adenocarcinoma Anticancer Res 2005 25 4069 74 16309200

[B18] Calcagno DQ Leal MF Seabra AD Khayat AS Chen ES Demachki S Assumpção PP Faria MH Rabenhorst SH Ferreira MV Interrelationship between chromosome 8 aneuploidy, *C-MYC *amplification and increased expression in individuals from northern Brazil with gastric adenocarcinoma World J Gastroenterol 2006 12 6207 11 17036397 10.3748/wjg.v12.i38.6207 PMC4088119

[B19] Guimarães AC Quintana LG Leal MF Takeno SS Assumpção PP Lima EM Khayat AS Chen ES Smith MdeAC Burbano RR Aneuploidy of chromosome 8 detected by fluorescence in situ hybridisation in ACP01 cell line gastric adenocarcinomas Clin Exp Med 2006 6 129 33 10.1007/s10238-006-0108-5 17061062

[B20] Panani AD Cytogenetic and molecular aspects of gastric cancer: clinical implications Cancer Lett 2008 266 99 115 10.1016/j.canlet.2008.02.053 18381231

[B21] Kobayashi M Kawashima A Mai M Ooi A Analysis of chromosome 17p13 (p53 locus) alterations in gastric carcinoma cells by dual-color fluorescence in situ hybridization Am J Pathol 1996 149 1575 84 8909247 PMC1865272

[B22] Takahashi Y Nagata T Asai S Shintaku K Eguchi T Ishii Y Fujii M Ishikawa K Detection of aberrations of 17p and p53 gene in gastrointestinal cancers by dual (two-color) fluorescence in situ hybridization and GeneChip p53 assay Cancer Genet Cytogenet 2000 121 38 43 10.1016/S0165-4608(00)00231-4 10958939

[B23] Gomyo Y Osaki M Kaibara N Ito H Numerical aberration and point mutation of p53 gene in human gastric intestinal metaplasia and well-differentiated adenocarcinoma: analysis by fluorescence in situ hybridization (FISH) and PCR-SSCP Int J Cancer 1996 66 594 9 10.1002/(SICI)1097-0215(19960529)66:5<594::AID-IJC2>3.0.CO;2-O 8647618

[B24] Suzuki S Tenjin T Shibuya T Tanaka S Chromosome 17 copy numbers and incidence of p53 gene deletion in gastric cancer cells. Dual color fluorescence in situ hybridization analysis Nippon Ika Daigaku Zasshi 1997 64 22 9 9119949 10.1272/jnms1923.64.22

[B25] Szymanska K Hainaut P *TP53 *and mutations in human cancer Acta Biochim Pol 2003 50 231 8 12673364

[B26] Fenoglio-Preiser CM Wang J Stemmermann GN Noffsinger A *TP53 *and gastric carcinoma: a review Hum Mutat 2003 21 258 70 10.1002/humu.10180 12619111

[B27] Hurt EM Thomas SB Peng B Farrar WL Reversal of p53 epigenetic silencing in multiple myeloma permits apoptosis by a p53 activator Cancer Biol Ther 2006 5 1154 60 10.1158/1535-7163.MCT-05-0446 16855375

[B28] Batista dos Santos SE Rodrigues JD Ribeiro-dos-Santos AK Zago MA Differential Contribution of Indigenous Men and Women to the Formation of an Urban Population in the Amazon Region as Revealed by mtDNA and y-DNA Am J Phys Anthropol 1999 109 175 80 10.1002/(SICI)1096-8644(199906)109:2<175::AID-AJPA3>3.0.CO;2-# 10378456

[B29] Laurén P The two histological main types of gastric carcinoma: diffuse and so-called intestinal-type carcinoma. An attempt at a histo-clinical classification Acta Pathol Microbiol Scand 1965 64 31 49 14320675 10.1111/apm.1965.64.1.31

[B30] Sobin LH Wittekind CH eds TNM: classification of malignant tumours 2002 6 New York: Wiley-Liss

[B31] Pinkel D Straume T Gray JW Cytogenetic analysis using quantitative, high-sensitivity, fluorescence hybridization Proc Natl Acad Sci USA 1986 83 2934 8 3458254 10.1073/pnas.83.9.2934 PMC323421

[B32] Hopman AH Ramaekers FC Raap AK Beck JL Devilee P Ploeg M van der Vooijs GP In situ hybridization as a tool to study numerical chromosome aberrations in solid bladder tumors Histochemistry 1988 89 307 16 10.1007/BF00500631 3410743

[B33] Ozturk Y Ozer E Lebe B Bekem O Buyukgebiz B Immunohistochemical evaluation of p53 expression and proliferative activity in children with Helicobacter pylori associated gastritis J Pediatr Gastroenterol Nutr 2005 40 467 70 10.1097/01.MPG.0000148832.22130.D7 15795596

[B34] Duensing A Duensing S Guilt by association? p53 and the development of aneuploidy in cancer Biochem Biophys Res Commun 2005 331 694 700 10.1016/j.bbrc.2005.03.157 15865924

[B35] Takeno SS Leal MF Lisboa LC Lipay MV Khayat AS Assumpção PP Burbano RR Smith Mde A Genomic alterations in diffuse-type gastric cancer as shown by high-resolution comparative genomic hybridization Cancer Genet Cytogenet 2009 190 1 7 10.1016/j.cancergencyto.2008.09.007 19264226

[B36] Burbano RR Assumpção PP Leal MF Calcagno DQ Guimarães AC Khayat AS Takeno SS Chen ES De Arruda Cardoso Smith M C-MYC locus amplification as metastasis predictor in intestinal-type gastric adenocarcinomas: CGH study in Brazil Anticancer Res 2006 26 2909 14 16886612

[B37] Galipeau PC Cowan DS Sanchez CA Barrett MT Emond MJ Levine DS Rabinovitch PS Reid BJ 17p (p53) allelic losses, 4N (G2/tetraploid) populations, and progression to aneuploidy in Barrett's esophagus Proc Natl Acad Sci USA 1996 93 7081 4 8692948 10.1073/pnas.93.14.7081 PMC38939

[B38] Williams L Jenkins GJ Doak SH Fowler P Parry EM Brown TH Griffiths AP Williams JG Parry JM Fluorescence *in situ *hybridisation analysis of chromosomal aberrations in gastric tissue: the potential involvement of Helicobacter pylori Br J Cancer 2005 92 1759 66 10.1038/sj.bjc.6602533 15827559 PMC2362026

[B39] César AC Borim AA Caetano A Cury PM Silva AE Aneuploidies, deletion, and overexpression of TP53 gene in intestinal metaplasia of patients without gastric cancer Cancer Genet Cytogenet 2004 153 127 32 10.1016/j.cancergencyto.2004.01.017 15350302

[B40] Gabbert HE Müller W Schneiders A Meier S Hommel G The relationship of p53 expression to the prognosis of 418 patients with gastric carcinoma Cancer 1995 76 720 6 10.1002/1097-0142(19950901)76:5<720::AID-CNCR2820760503>3.0.CO;2-E 8625172

[B41] Poremba C Yandell DW Huang Q Little JB Mellin W Schmid KW Böcker W Dockhorn-Dworniczak B Frequency and spectrum of p53 mutations in gastric cancer–a molecular genetic and immunohistochemical study Virchows Arch 1995 426 447 55 10.1007/BF00193167 7633655

[B42] Kim JH Uhm HD Gong SJ Shin DH Choi JH Lee HR Noh SH Kim BS Cho JY Rha SY Relationship between p53 overexpression and gastric cancer progression Oncology 1997 54 166 70 10.1159/000227682 9075790

[B43] Gürel S Dolar E Yerci O Samli B Oztürk H Nak SG Gülten M Memik F Expression of p53 protein and prognosis in gastric carcinoma J Int Med Res 1999 27 85 9 10446695 10.1177/030006059902700205

[B44] Lee DY Park CS Kim HS Kim JY Kim YC Lee S Maspin and p53 protein expression in gastric adenocarcinoma and its clinical applications Appl Immunohistochem Mol Morphol 2008 16 13 8 18091326 10.1097/PAI.0b013e31802c4f21

